# Deep Learning in Prostate Cancer Diagnosis Using Multiparametric Magnetic Resonance Imaging With Whole-Mount Histopathology Referenced Delineations

**DOI:** 10.3389/fmed.2021.810995

**Published:** 2022-01-13

**Authors:** Danyan Li, Xiaowei Han, Jie Gao, Qing Zhang, Haibo Yang, Shu Liao, Hongqian Guo, Bing Zhang

**Affiliations:** ^1^Department of Radiology, Nanjing Drum Tower Hospital Clinical College of Nanjing Medical University, Nanjing, China; ^2^Department of Radiology, Nanjing Drum Tower Hospital, The Affiliated Hospital of Nanjing University Medical School, Nanjing, China; ^3^Department of Urology, Nanjing Drum Tower Hospital, The Affiliated Hospital of Nanjing University Medical School, Nanjing, China; ^4^Department of Research and Development, Shanghai United Imaging Intelligence Co., Ltd., Shanghai, China

**Keywords:** prostate cancer, deep learning, magnetic resonance imaging, segmentation, detection

## Abstract

**Background:** Multiparametric magnetic resonance imaging (mpMRI) plays an important role in the diagnosis of prostate cancer (PCa) in the current clinical setting. However, the performance of mpMRI usually varies based on the experience of the radiologists at different levels; thus, the demand for MRI interpretation warrants further analysis. In this study, we developed a deep learning (DL) model to improve PCa diagnostic ability using mpMRI and whole-mount histopathology data.

**Methods:** A total of 739 patients, including 466 with PCa and 273 without PCa, were enrolled from January 2017 to December 2019. The mpMRI (T2 weighted imaging, diffusion weighted imaging, and apparent diffusion coefficient sequences) data were randomly divided into training (*n* = 659) and validation datasets (*n* = 80). According to the whole-mount histopathology, a DL model, including independent segmentation and classification networks, was developed to extract the gland and PCa area for PCa diagnosis. The area under the curve (AUC) were used to evaluate the performance of the prostate classification networks. The proposed DL model was subsequently used in clinical practice (independent test dataset; *n* = 200), and the PCa detective/diagnostic performance between the DL model and different level radiologists was evaluated based on the sensitivity, specificity, precision, and accuracy.

**Results:** The AUC of the prostate classification network was 0.871 in the validation dataset, and it reached 0.797 using the DL model in the test dataset. Furthermore, the sensitivity, specificity, precision, and accuracy of the DL model for diagnosing PCa in the test dataset were 0.710, 0.690, 0.696, and 0.700, respectively. For the junior radiologist without and with DL model assistance, these values were 0.590, 0.700, 0.663, and 0.645 versus 0.790, 0.720, 0.738, and 0.755, respectively. For the senior radiologist, the values were 0.690, 0.770, 0.750, and 0.730 vs. 0.810, 0.840, 0.835, and 0.825, respectively. The diagnosis made with DL model assistance for radiologists were significantly higher than those without assistance (*P* < 0.05).

**Conclusion:** The diagnostic performance of DL model is higher than that of junior radiologists and can improve PCa diagnostic accuracy in both junior and senior radiologists.

## Introduction

Prostate cancer (PCa) is a major public health problem, representing the most common cancer type and the second highest cancer mortality among men in western countries (1). Multiparametric magnetic resonance imaging (mpMRI) plays an important role in diagnosis, targeted puncture guidance, and prognosis assessment of PCa in the current clinical setting (2). However, the performance of mpMRI usually varies based on the experience of radiologists at different levels (3), and the demand for MRI interpretation is ever-increasing. A convolutional neural network (CNN) approach, which can surpass human performance in natural image analysis, is anticipated to enhance computer-assisted diagnosis in prostate MRI (4, 5).

The CNN-based deep learning (DL) method revolutionizes and reshapes the existing work pattern. Diffusion weighted imaging (DWI), apparent diffusion coefficient (ADC), and T2-weighted imaging (T2WI) sequences are probably the most important and practical components of clinical prostate MRI examinations (6, 7). Several previous studies on DL involved a PCa diagnosis using only one or two of the above sequences and thus cannot be directly compared with clinical performance (8, 9).

Some studies focused on DL models with MRI data labeling based on biopsy locations that were determined by radiologists (10), which could result in inaccurate labeling. Whole-mount tissue sections, in which the entire cross-section of tissue from the gross section is mounted to the slide, provide pathologists with a good overview facilitating the identification of tumor foci (11–13). The use of prostate specimen whole-mount sectioning provides significantly superior anatomical registration for PCa than just mpMRI. Herein, we propose that the radiologists label PCa lesions on the MRI images using whole-mount histopathology images as reference to increase the accuracy of the labels.

In this study, a DL method was proposed to automatically conduct prostate gland segmentation, classification, and regional segmentation of PCa lesions, and subsequently compare its diagnostic efficiency with different level radiologists in clinical practice.

## Materials and Methods

This retrospective study was approved by the Ethics Institution of Nanjing Drum Tower Hospital, and informed consent was waived since T2WI, DWI, and ADC sequences are part of the routine protocols for prostate MRI scans.

### Patients

**Figure 1:**
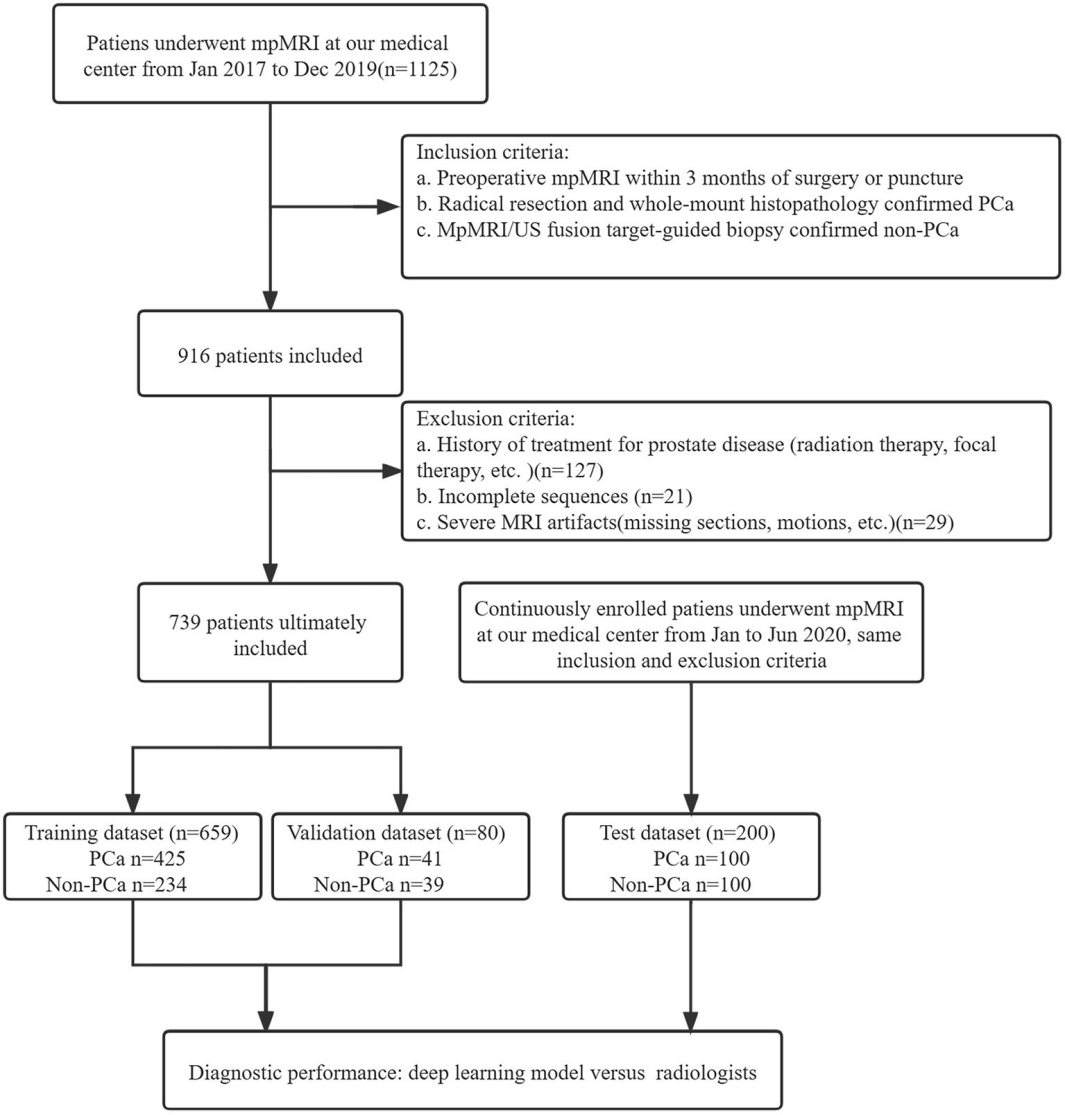
Study flowchart of patient selection. PSA, prostate-specific antigen; mpMRI, multiparametric MRI; US, ultrasound.

A total of 1125 patients who underwent prostate mpMRI between January 2017 and December 2019 were enrolled in the study. The inclusion criteria were as follows: (a) preoperative mpMRI within 3 months of surgery or puncture, (b) radical resection and whole-mount histopathology-confirmed PCa, and (c) mpMRI/ultrasonography (US) fusion target-guided biopsy or surgery confirmed non-PCa. Patients without PCa were defined as having negative biopsy or surgery. The exclusion criteria were (a) a history of treatment for prostate disease (radiation therapy, focal therapy, etc.), (b) incomplete imaging sequences, (c) severe MRI artifacts (missing sections, motions, etc.), and (d) unavailable whole-mount history. All MRI scans were reviewed in consensus by two radiologists, a 5-year junior and 10-year senior radiologist specializing in genitourinary imaging. A total of 739 patients, including 466 patients with PCa and 273 patients without PCa, were included for training and validation in the model. The independent dataset was consecutively collected from January 2020 to June 2020 with the same inclusion and exclusion criteria as mentioned above. A flowchart of the patient selection is shown in Figure 1.

### MR Imaging

Patients were scanned using two 3.0 T MRI scanners (uMR770; United Imaging, Shanghai, China and Ingenia; Philips Healthcare, Best, the Netherlands) with the same sequences and standard phased array surface coils according to the European Society of Urogenital Radiology guidelines. T_1_WI, T_2_WI, DWI and ADC sequences were acquired. Detailed parameters for transverse DWI (b-values of 50, 1,000, and 1,500 s/mm^2^) were as follows: repetition time (TR), 5,100 ms; echo time (TE), 80 ms; field of view, 26 × 22 cm; and thickness, 3 mm. Low b-value images were acquired at 50 s/mm^2^ to avoid perfusion effects at a b-value of 0 s/mm^2^. ADC maps were calculated from the b-value (1,500 s/mm^2^) using the scanner software. T_2_WI, DWI (b-values of 1,500 s/mm^2^), and ADC (b-values of 1,500 s/mm^2^) sequences were used in this study.

### Histopathology

All the cases were confirmed by mpMRI/US fusion-guided targeted biopsy, and patients with PCa were further confirmed by radical resection and whole-mount histopathology. All the biopsies were conducted using the MRI-trans rectal ultrasound scan (TRUS) image registration system (Esaote® and RVS®). Whole-mount specimens were sliced from the apex to the base at 3-mm intervals following prostatectomy. All the specimens were examined by two independent urological pathologists.

### Prostate Gland and Cancer Region Labeling Referenced by Whole-Mount Histopathology Image in the Training and Validation Datasets

**Figure 2:**
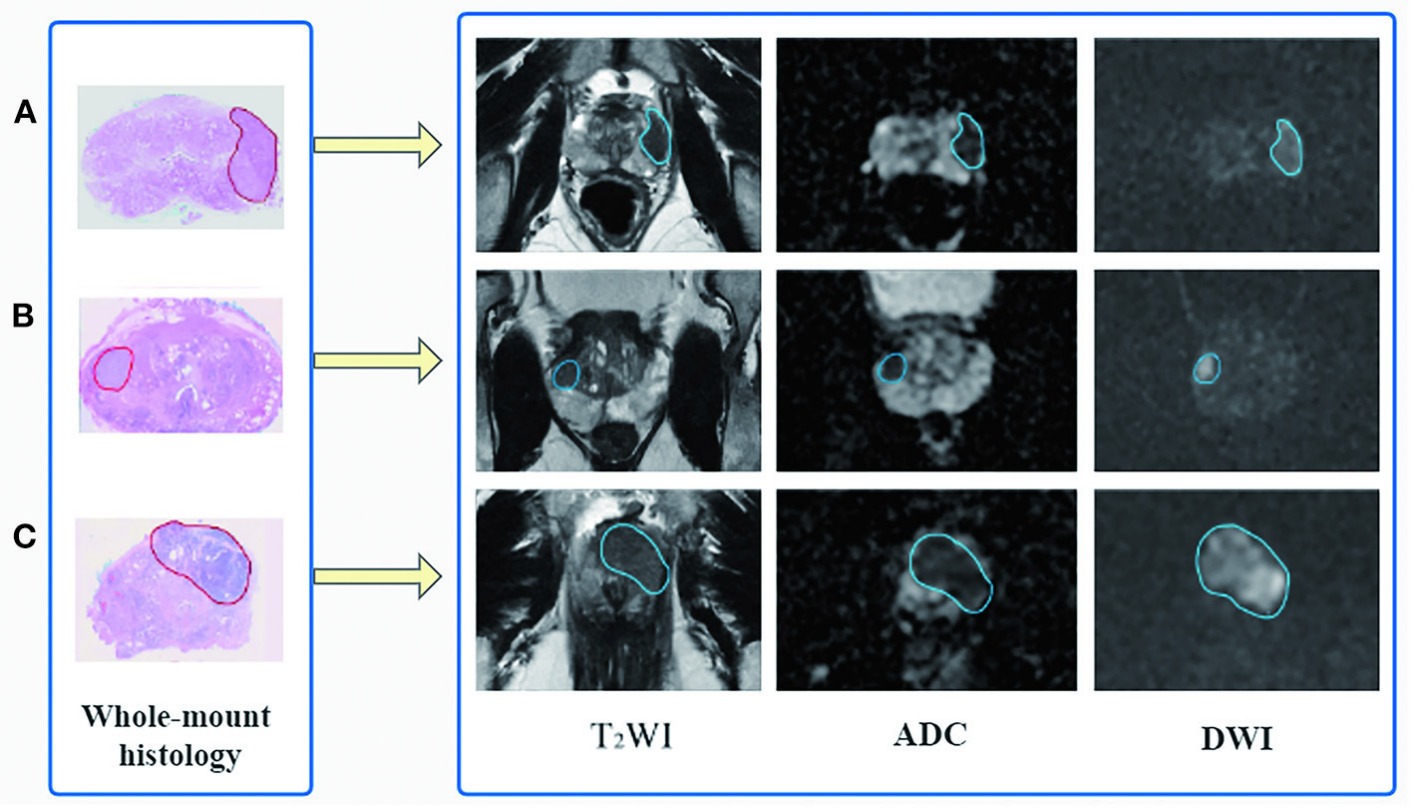
Flowchart of region of interest delineation for prostate cancer lesion. All the prostate cancer lesions were manually labeled on the magnetic resonance images using whole-mount histopathology as a reference. Representative cases of prostate cancer in different zone distributions: **(A)** the lesion is in the left peripheral zone, **(B)** in the right peripheral and transition zone, and **(C)** in the transition zone and anterior fibromuscular stroma.

Based on the whole-mount histopathology images, the prostate gland and all the cancer regions on T2WI, DWI, and ADC sequences were labeled by two radiologists (with 5 and 10 years of expertise, respectively) under the supervision of a superior radiologist (with 15 years of expertise) using the open-source software ITK-SNAP (http://www.simpleitk.org, version 3.8.0). The workflow is illustrated in Figure 2.

### The DL Network Structure

**Figure 3:**
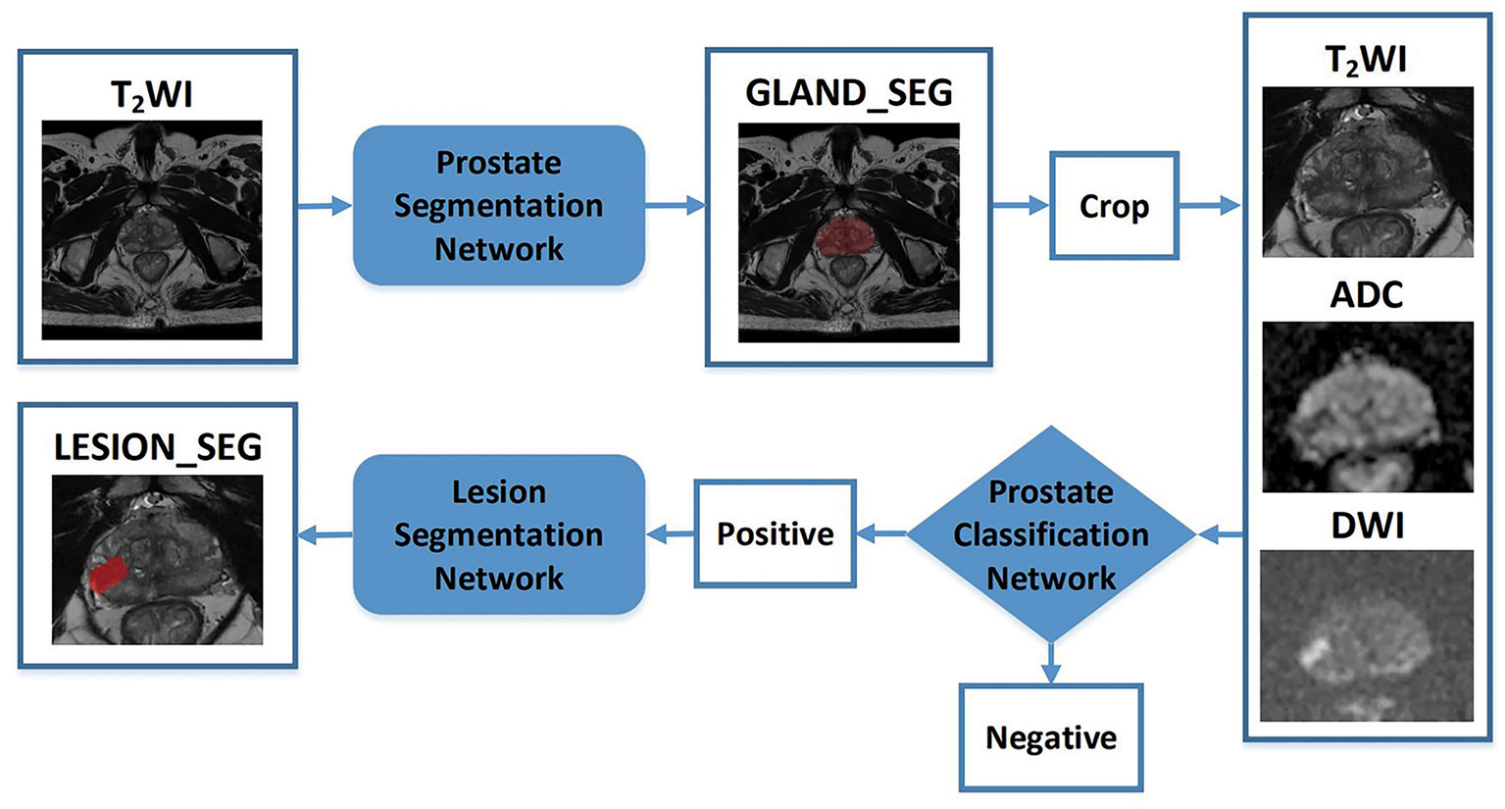
Flowchart of the study. The blocks highlighted in blue (prostate gland segmentation network, prostate cancer classification network, prostate cancer segmentation/detection network) denote network models used in our study. “Crop” represents a fixed size region of interest (ROI) to crop the prostate gland according the result of the prostate gland segmentation network. The cropped ROI of ADC and DWI would be registered to the cropped ROI of the T2-weighted imaging (T2WI) and then three cropped ROI would be fed into the prostate cancer classification network. “Positive” represents the positive output of the classification network; in that case, the cropped ROI would be fed into the prostate cancer segmentation network to obtain the lesion region. “Negative” represents the negative output of the classification network; in that case, the cropped ROI would not be fed into the prostate cancer segmentation network.

A CNN was constructed for prostate gland segmentation, classification, and cancer region segmentation/detection tasks. The model structure is illustrated in Figure 3.

First, a prostate gland segmentation network based on the T2WI sequence was implemented to obtain a mask of the gland. The mask was subsequently cropped to obtain three image patches including the gland on T2WI, DWI, and ADC sequences. Second, a prostate classification network based on the image patches from the T2WI, DWI, and ADC sequences was used to determine whether the gland had PCa lesion(s). If the gland was abnormal, a PCa segmentation network was used to obtain the lesion region. It is worth noting that the T2WI, DWI, and ADC patches were obtained based on the prostate gland segmentation results and were of fixed and similar sizes, including the gland.

The prostate gland segmentation network was based on V-Net (14), as shown in Supplementary Figure 1. The classification network was based on dense convolutional network (DenseNet) (15), which was used to determine whether the gland was normal. DenseNet connects each layer to every other layer in a feed-forward fashion. The feature maps of all the preceding layers were used as inputs for each layer, and their feature maps were used as inputs to all the subsequent layers. Prostate cancer lesion segmentation was also performed based on the image patches of T2WI, DWI, and ADC sequences. To obtain a more accurate cancer region, the Up-Block in V-Net was changed to an Up SE-Block, which adds a squeeze-and-excitation operation following two convolutions, as shown in Supplementary Figure 2.

### Training and Optimization Details

In this study, all the networks were implemented using the PyTorch framework and Python 3.7. All the learning computations were performed on a Tesla V100 DGXS GPU with 32 GB of memory. The adaptive moment (Adam) algorithm was applied to optimize the parameters of the prostate segmentation network. The training dataset was randomly shuffled, and a batch size of four was selected. The stochastic gradient descent (SGD) algorithm was applied to optimize the parameters of the PCa network (15). The training dataset was randomly shuffled, and a batch size of 12 was selected. Finally, the Adam algorithm was applied again to optimize the parameters of the PCa region segmentation network. The training dataset was randomly shuffled, and a batch size of four was selected. During the training process for the prostate gland and cancer segmentation networks, the Dice loss was adopted, and the network weights were updated using the Adam optimizer with an initial learning rate of 0.0001. During the training process for the classification network, the cross-entropy loss was adopted, and the network weights were updated using SGD with an initial learning rate of 0.1.

### Image Analysis of the Junior and Senior Radiologists for the Test Dataset Without and With DL Assistance

The T2WI, DWI, and ADC images were imported from the DICOM format into ITK-SNAP (version 3.8.0). The MR images with and without DL delineations were independently reviewed by two radiologists, a 5-year junior and 10-year senior radiologist specializing in genitourinary imaging, who were blinded to the pathological results. PI-RADS v2.1 (16) recommendations were used by the junior and senior radiologists to evaluate the PCa likelihood of suspicious areas on mpMRI for each patient (17), and the results were divided into PCa (PI-RADS score 4-5 and partly PI-RADS score 3 cases) and non-PCa. Particularly, referring to PI-RADS score 3 cases, the final diagnosis would be further made by another 20-year radiologist specializing in genitourinary imaging.

### Statistical Analysis

Continuous variables are described using mean ± standard deviation, while categorical variables are described using frequency and ratio. The chi-square test was used for the sample size and location distribution. The DL model was verified using the validation and test datasets. The Dice loss was used to evaluate the performance of prostate gland and PCa lesion segmentation networks. The cross-entropy loss and AUC were used to evaluate the performance of the classification networks. Furthermore, the sensitivity, specificity, precision and accuracy were used to evaluate the diagnostic performance of the model in clinical application.

## Results

### Study Sample Characteristics

Patient demographic data and characteristics of the training, validation, and test datasets are shown in Table 1. There were no significant differences in the patient age or total prostate-specific antigen (PSA) values among the three groups. In the training dataset, there were 500 pathologically proven cancer lesions, with 315 lesions in the peripheral zone (PZ), 146 in the transitional zone (TZ), 3 in the anterior fibromuscular stroma (AFS), and 36 in the mixed region. In the validation data set, there were 59 pathologically proven cancer lesions, with 42 lesions in the PZ, 10 in the TZ, 0 in the AFS, and 7 in the mixed region. In the test dataset, there were 127 pathologically proven cancer lesions, with 78 lesions in the PZ, 38 in the TZ, 1 in the AFS, and 10 in the mixed region.

**Table 1:** Clinical and imaging characteristics of the included patients.

**Characteristics**	**Training dataset**	**Validation dataset**	**Test dataset**	**F/χ^2^**	***P***
	***n* = 659**	***n* = 80**	***n* = 200**		
Age, mean ± SD(y)	68.1 ± 7.8	67.5 ± 5.4	67.7 ± 7.4	0.53	0.58
Prostate cancer, *n* (%)	425 (64.5)	41 (51.3)	100 (50)	17.22	<0.01
Non prostate cancer, *n* (%)	234 (35.5)	39 (48.7)	100 (50)		
tPSA level (ng/ml)	17.8 ± 22.2	14.9 ± 15.7	15.9 ± 22.1	0.91	0.40
Prostate cancer	15.3 ± 21.3	22.0 ± 23.1	13.1 ± 17.7	0.04	0.96
Non prostate cancer	9.0 ± 5.3	7.35 ± 4.7	9.1 ± 6.4	1.18	0.31
Prostate cancer lesion numbers	500	59	127		
Prostate cancer zone distribution, n (%)					
PZ	315 (63.0)	42 (71.2)	78 (61.4)	5.37	0.49
TZ	146 (29.2)	10 (16.9)	38(29.9)		
AFS	3 (0.6)	0	1 (0.8)		
Mixed	36 (7.2)	7 (11.9)	10 (7.9)		

*The data are reported as the mean ± standard deviations*.

*PSA, prostate-specific antigen; PZ, peripheral zone; TZ, transitional zone; AFS, anterior fibromuscular stroma*.

### Performance of the DL Model in the Training Dataset

The training epoch was set as 700 for the prostate gland segmentation network, while the Dice loss values converged to 0.068; the convergence graph is shown in Supplementary Figure 3. A total of 330 epochs were set for training the prostate classification model, and the cross-entropy loss converged to 0.120; the convergence graph is shown in Supplementary Figure 4. For the PCa segmentation model, the network was trained for 240 epochs, when the value of the loss function converged to 0.167. A convergence graph is shown in Supplementary Figure 5.

### Performance of the DL Model in the Validation Dataset

**Figure 4:**
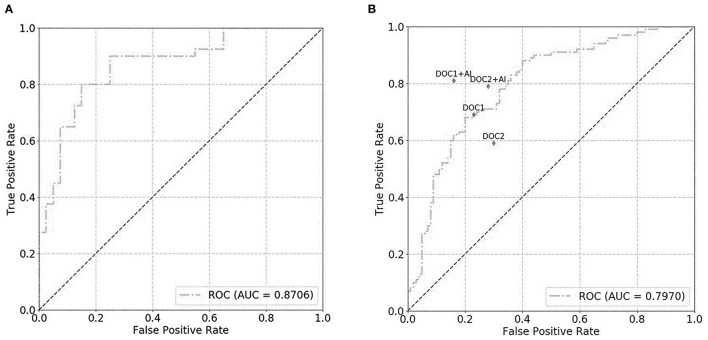
The graph shows the receiver operating characteristic (ROC) curve for prostate classification network performance. The ROC curves for validation set **(A)** and test set **(B)** show area under the curve (AUC) of 0.871 and 0.797, respectively. DOC1, senior radiologist; DOC2, junior radiologist.

For the prostate gland automatic segmentation efficacy, the Dice loss values converged to 0.076, and the convergence graph is shown in Supplementary Figure 3. For the prostate automatic classification efficacy, the cross-entropy loss converged to 0.224, and the convergence graph is shown in Supplementary Figure 4. The AUC value for the prostate classification network was 0.871 (Figure 4A). For the prostate cancer automatic segmentation efficacy, the Dice loss values converged to 0.484, as shown in Supplementary Figure 5.

### Diagnostic Performance of Prostate Cancer by Different Radiologists and DL Model in the Test Dataset

**Figure 5:**
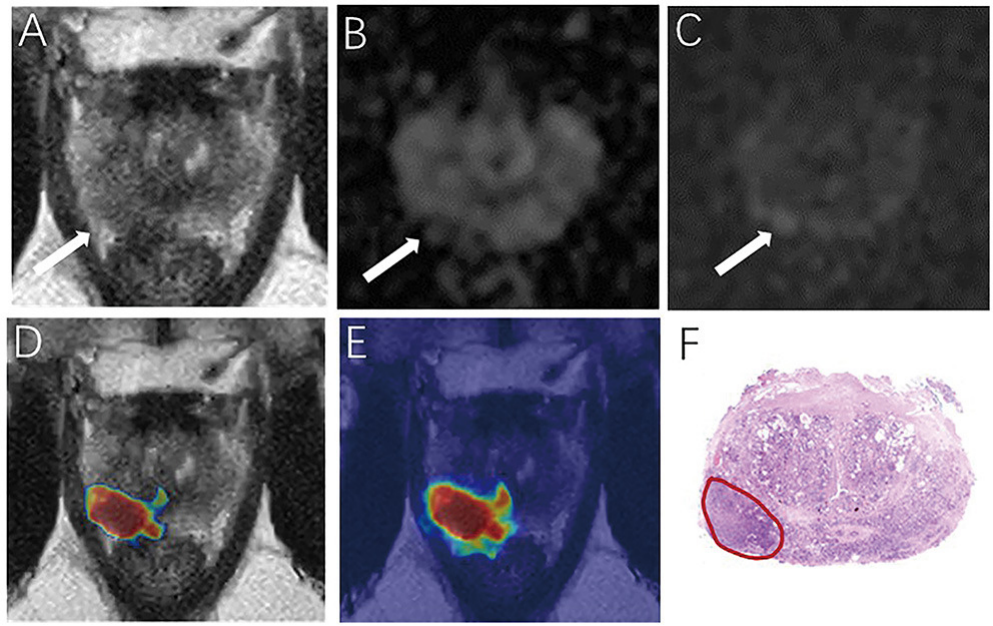
Demonstrate representative prostate cancer (PCa) example of radiologists negative **(A–C)** and deep learning (DL) model positive **(D,E)**. Images show a case of DL model segmentation in a 60-year patient in a test set with prostate-specific antigen (PSA) of 5.59 ng/mL. Axial T2-weighted image **(A)** shows an ill-defined area of little low signal in the right peripheral zone (arrow), with slight restricted diffusion on apparent diffusion coefficient (ADC) maps **(B)**. **(C)** Diffusion weighted imaging (DWI) (b-value 1,500 sec/mm^2^) shows slightly increased signal in this region, with an obvious conspicuity over background normal signal; this lesion would be PI-RADS score 3 for magnetic resonance imaging (MRI). **(D,E)** show overlapping areas between DL focused PCa region and genuine cancer location. The overlapped areas are colored in red. The software ITK-SNAP was used to open the probability map and MR images at the same time. Through the software function, the probability map is displayed as a jet type color map and overlappedon the T2 weighted imaging (T2WI) to obtain **(E)**; The window width and window level of the probability map is adjusted to 0.5 and 0.75 respectively to display the probability map of the detected cancer area and overlapped on the image to obtain **(D)**.

For the prostate automatic classification efficacy, the cross-entropy loss converged to 0.236. The AUC value for the prostate classification network was 0.797 in the test dataset (Figure 4B). Table 2 shows the evaluation of the model's diagnostic efficiency in practical applications based on the sensitivity, specificity, precision, and accuracy, with values of 0.710, 0.690, 0.696, and 0.700, respectively. For the junior radiologist without and with DL model assistance, these values were 0.590, 0.700, 0.663, and 0.645 vs. 0.790, 0.720, 0.738, and 0.755, respectively. For the senior radiologist, the values were 0.690, 0.770, 0.750, and 0.730 vs. 0.810, 0.840, 0.835, and 0.825, respectively. The values obtained with DL model assistance for radiologists were significantly higher than those without assistance (*P* < 0.05). Figure 5 shows a representative PCa example of radiologist-negative but DL model positive.

**Table 2:** Diagnostic performance of prostate cancer by different radiologists and DL model.

**Group**	**Sensitivity**	**Specificity**	**Precision**	**Accuracy**
Junior radiologist	0.590	0.700	0.663	0.645
Senior radiologist	0.690	0.770	0.750	0.730
DL model	0.710	0.690	0.696	0.700
DL+Junior	0.790	0.720	0.738	0.755
DL+Senior	0.810	0.840	0.835	0.825

*Junior and senior radiologist, experienced in interpreting prostate MRI (5 and 10 years, respectively); DL, deep learnig*.

## Discussion

We proposed a DL model for improving the diagnostic ability of PCa using mpMRI and whole-mount histopathology images referenced delineations. The DL model diagnostic ability was higher than that of a junior radiologist and can improve PCa diagnostic accuracy in both junior and senior radiologists in clinical practice.

MpMRI plays an important role in the diagnostic workflow of patients with suspected PCa (18). DWI, ADC, and T2WI are probably the most important sequences in the detection, identification, and staging of PCa (19–21), and the DCE sequence offers limited added value compared to T2+ADC+DWI (22). According to PI-RADS V2.1, the role of the DCE sequence is only helpful for score 3 lesions in the PZ (7). Some study also observed that for DWI score 3 lesions in the PZ of biopsy-negative patients, the DCE sequence had no significant increased value in improving the identification of PCa (13). So, we proposed a DL model based on DWI, ADC, and T2WI sequences without contrast medium injections. Furthermore, some previous studies on DL model using only one or two of the above sequences and thus cannot be directly compared with clinical performance (8, 9).

The use of whole-mount histopathological specimens is a strong reference standard. Moreover, Padhani et al. (23) suggested that training datasets with spatially well-correlated histopathologic validation should be used. Our previous studies confirmed that whole-mount sections can be used as a reference to obtain a highly accurate prostate lesion label on prostate mpMRI (13). We subsequently labeled the PCa lesions on MR images using whole-mount histopathology images as references. Furthermore, the DL model was developed based on the classic V-Net and DenseNet networks; SE-Block integrated variables controlling also helped in improving the model accuracy and performance (24). In our study, the DL model was used to extract the gland and PCa areas, and accurately identify PCa compared to the gold standard of histopathology. The AUC value was 0.797 for the prostate classification network in the test dataset, and the accuracy of PCa detection/diagnosis was 0.700, which is higher than that of several reports. For example, Ishioka et al. (25) proposed a CNN deep learning model with AUCs of 0.645 and 0.636 in two validation sets, respectively. Moreover, our independent test dataset is imported without gland or lesion labeling in order to evaluate the model in real clinical work scenarios. The average PSA level of non-PCa group in the test dataset was 9.1 ± 6.4 ng/ml. It was a little high because all the patients were confirmed by targeted biopsy or resection for prostatitis, hyperplasia, or other prostate benign diseases; thus, the differential diagnosis could be challenging (26).

Castillo et al. (27) systematically reviewed the performance of machine learning applications in PCa classification based on MRI, and found that only one paper (27 publications) compared the performance of radiologists with or without DL model assistance, and presented that evaluation should be performed in a real clinical setting since the ultimate goal of these models is to assist the radiologists in diagnosis. Seetharaman et al. (28) developed a SPCNet model accurately detected aggressive PCa. In our study, we evaluated the DL model in an independent test dataset to assess its clinical application value and to compare it with junior and senior radiologists. This DL model showed higher accuracy than junior radiologist in diagnosing PCa and slightly lower than the senior radiologist. Furthermore, the DL model improved PCa diagnostic accuracy for both junior and senior radiologists. This is similar to the findings of Cao et al. (29), who presented a DL algorithm (FocalNet) that achieved slightly but not significantly lower PCa diagnosis performance than genitourinary radiologists. Additionally, some studies demonstrated diagnostic accuracy for prostate cancer using PI-RADS was 71.0 83.5%, which was similar with our results, but PI-RADS usually varies based on the experience of radiologists at different levels (30, 31).

Currently, most DL models are not fully automated diagnosis systems; rather, they are adjunct tools that aid radiologists in reading prostate mpMRI results. Kotter et al. (32) determined that new DL technology would not threaten a radiologist's career but rather help strengthen his or her diagnostic ability. In summary, our proposed DL model can improve PCa diagnostic performance for both senior and junior radiologists, indicating that DL assistance can potentially improve the clinical workflow.

### Limitations and Outlook

There are several limitations to this study. First, all the patients were recruited from a single center. This may have negatively affected the performance of the model because larger and more diverse patient groups improve the generalizability of the classification algorithms. Second, the study was retrospective, the clinical data and traditional image parameters were not used in this study. Future studies should focus on multicenter data, biomarkers, and optimized algorithms to produce more reliable models for improving diagnosis, staging, and recurrence prediction of PCa. At last, all the patients were scanned using two 3.0 T MRI scanners in this study. The DL model may not perform so well using images provided by different machines or by a machine with a lower magnetic field.

## Conclusion

In this study, we proposed an automated DL model for the segmentation and detection of PCa based on mpMRI and whole-mount histopathology referenced delineations. The diagnostic performance of DL model is higher than junior radiologist and could be capable of improving the diagnostic accuracy for both junior and senior radiologists and applying for young radiologist training.

## Data Availability Statement

The raw data supporting the conclusions of this article will be made available by the authors, without undue reservation.

## Ethics Statement

The studies involving human participants were reviewed and this retrospective study was approved by the Ethics Institution of Nanjing Drum Tower Hospital. Written informed consent for participation was not required for this study in accordance with the national legislation and the institutional requirements.

## Author Contributions

DL and XH acquired and analyzed data and drafted the manuscript. HY and SL analyzed and explained the imaging data. JG and QZ acquired the clinical information and revised the manuscript. HG and BZ designed the study and revised the manuscript. All authors contributed to the article and approved the submitted version.

## Conflict of Interest

HY and SL were employed by company Shanghai United Imaging Intelligence Co. The remaining authors declare that the research was conducted in the absence of any commercial or financial relationships that could be construed as a potential conflict of interest.

## Publisher's Note

All claims expressed in this article are solely those of the authors and do not necessarily represent those of their affiliated organizations, or those of the publisher, the editors and the reviewers. Any product that may be evaluated in this article, or claim that may be made by its manufacturer, is not guaranteed or endorsed by the publisher.
