# Supplementary material for: Deep Learning in Prostate Cancer Diagnosis Using Multiparametric Magnetic Resonance Imaging With Whole-Mount Histopathology Referenced Delineations
Source: Front Med (Lausanne). 2022 Jan 13;8:810995. doi: 10.3389/fmed.2021.810995 (PMC8793798; doi:10.3389/fmed.2021.810995)
Supplement: Supplementary file 1 [file Data_Sheet_1.docx]

Supplementary Material

**Supplementary Figure 1. Prostate Segmentation Network.**


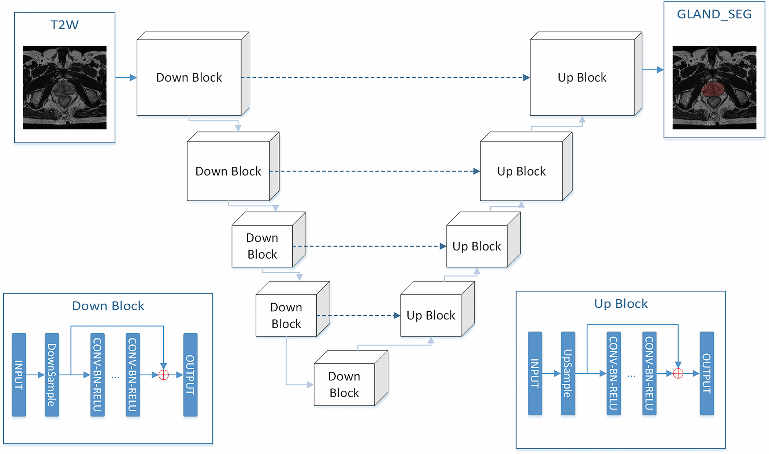


Architecture of prostate segmentation network: Prostate gland segmentation is based on T2 weighted imaging (T2WI) sequences. The main architecture of the network is based on V-Net. This network is composed of five down blocks and four up blocks. Each Down-Block consists of one residual block and one convolution layer with a 2 × 2 × 2 filter shape and stride of 2. The residual block has two convolution layers and one residual connection, and after each convolution batch normalization is added, the leaky rectified linear unit (ReLU) is used as the activation function. However, the convolution layer with a 2 × 2 × 2 filter shape and stride 2 in the first Down-Block was changed to 1 × 1 × 1 and 1. Each Up-Block consists of one residual block and one deconvolution layer with a 2 × 2 × 2 filter shape and stride of 2. The residual block is the same as that in the own block. The last block contains a softmax layer.

**Supplementary Figure 2. Lesion Segmentation Network.**


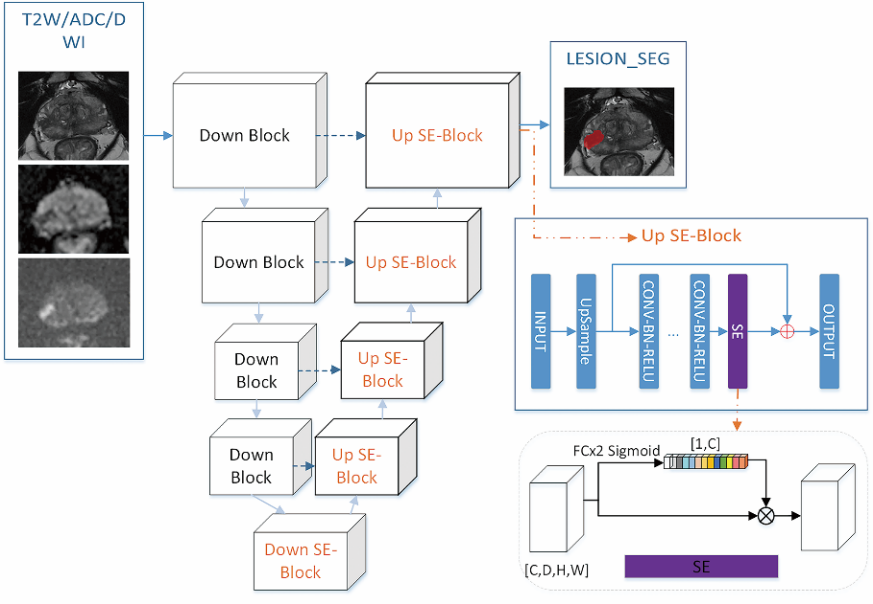


Architecture of lesion segmentation network: The prostate cancer region segmentation is based on T2 weighted imaging (T2WI), diffusion weighted imaging (DWI), and apparent diffusion coefficient (ADC) with Up SE-Block, which adds a squeeze-and-excitation operation after two convolutions. The squeeze-and-excitation operation uses global average pooling to squeeze global information into a channel descriptor and then uses two fully connected layers, followed by the rectified linear unit (ReLU) activation function, and a sigmoid function is used to capture channel-wise dependencies.

**Supplementary Figure 3. The prostate segmentation in the training and validation dataset.**


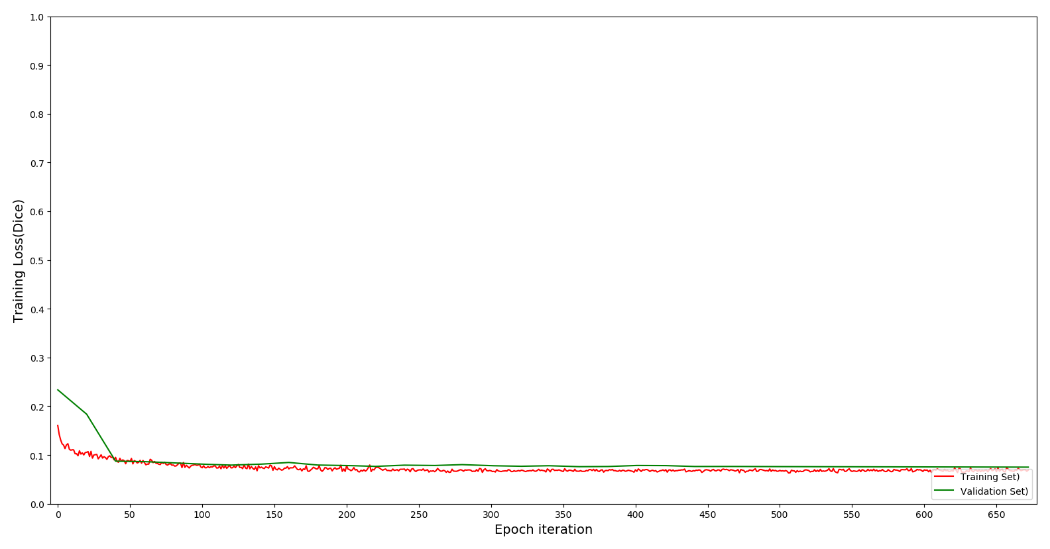


The adaptive moment (Adam) algorithm was applied to optimize the parameters of the prostate segmentation network. The training dataset was randomly shuffled, and a batch size of four was chosen. All the computations for learning were performed on a Tesla V100 DGXS GPU with 32 GB of memory. Dice loss was also adopted. The network was trained for 700 epochs when the value of the loss function converged to 0.068 in the training dataset. the Dice loss values converged to 0.076 in the validation dataset. The convergence graph is shown here.

**Supplementary Figure 4. The prostate classification in the training and validation dataset.**


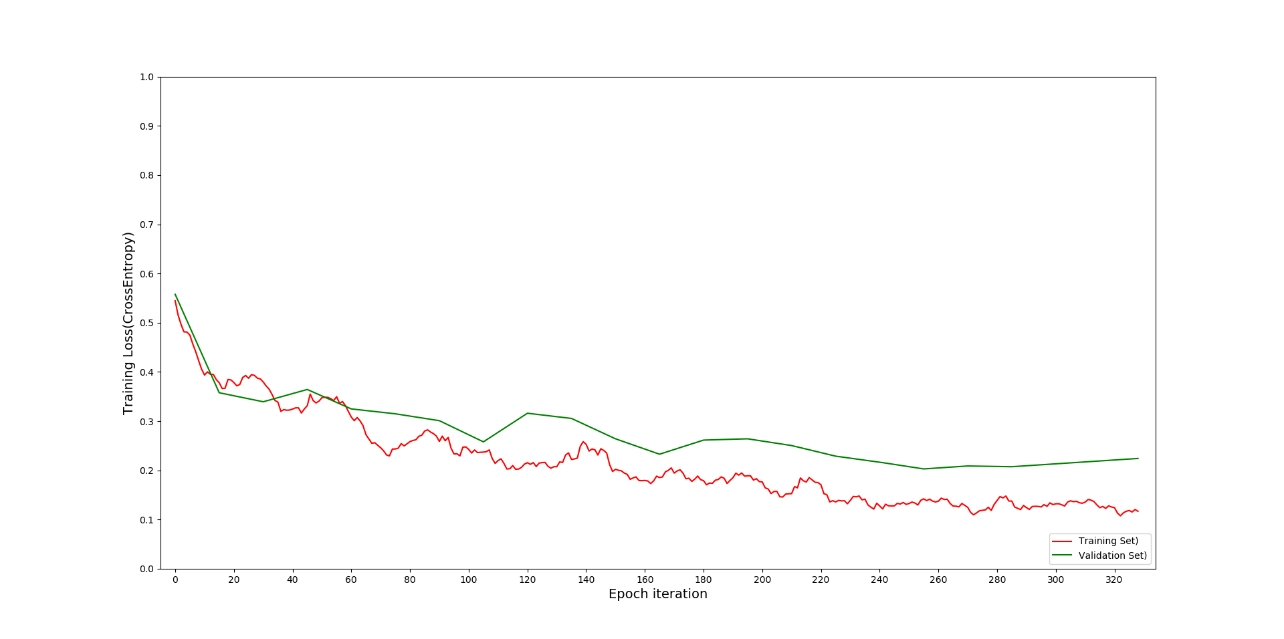


The stochastic gradient descent (SGD) algorithm was applied to optimize the parameters of the prostate classification network. The training dataset was randomly shuffled, and a batch size of 12 was chosen. All the computations for learning were performed on a Tesla V100 DGXS GPU with 32 GB of memory. The cross-entropy loss was adopted. The network was trained for 330 epochs when the value of the loss function converged to 0.120 in the training dataset. The cross-entropy loss converged to 0.224 in the validation dataset. The convergence graph is shown here.

**Supplementary Figure 5. The prostate cancer segmentation in the training and validation dataset.**


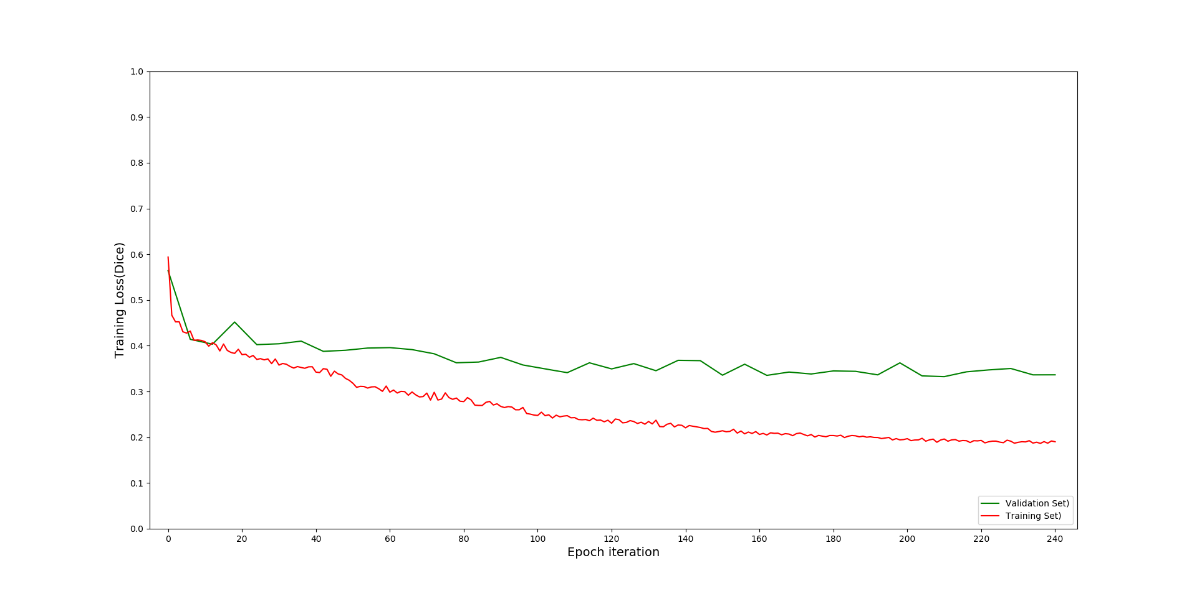


The adaptive moment (Adam) algorithm was applied to optimize the parameters of the prostate cancer region segmentation network. The training dataset was randomly shuffled, and a batch size of four was chosen. All computations for learning were performed on a Tesla V100 DGXS GPU with 32 GB of memory. Dice loss was also adopted. The network was trained for 240 epochs when the value of the loss function converged to 0.167 in the training dataset. The Dice loss values converged to 0.484 in the validation dataset. The convergence graph is shown here.
